# Hydroxytyrosol: biological activities and potential application in livestock production

**DOI:** 10.3389/fvets.2025.1632596

**Published:** 2025-11-24

**Authors:** Yang Gao, Wenhui Liu, Shengsheng Pan, Jiahui Li, Jingwei Wang, Lin Chen, Xue Ma, Huan Leng

**Affiliations:** 1 College of Life Science, Baicheng Normal University, Baicheng, China; 2 Zhangzhou Health Vocational College, Zhangzhou, China; 3 Terra Research and Teaching Centre, Microbial Processes and Interactions (MiPI), Gembloux Agro-Bio Tech, University of Liège, Gembloux, Belgium; 4 Key Laboratory of Development and Application of Rural Renewable Energy, Biogas Institute of Ministry of Agriculture and Rural Affairs, Chengdu, China

**Keywords:** hydroxytyrosol, livestock, gut health, oxidative stress, liver protection, feed additive

## Abstract

Hydroxytyrosol (HT) is a polyphenol compound that is widely present in the leaves and fruits of olive in the form of esters, which is one of the natural antioxidants. In recent years, a large number of studies have found that HT has good activity in anti-oxidant, anti-inflammatory, lipids lowering and other physiological functions. The purpose of this article is to provide solutions for the development of new feed additives under the premise of a complete ban on the use of antibiotics. In this review, we concluded the reports on HT in recent years and summarized its source, synthesis, digestion, absorption and metabolism of HT, as well as its main physiological functions, application prospects in animal production. Therefore, HT can be used as a potential new feed additive so as to provide theoretical basis and guidance for the development and application of hydroxytyrosol in animal husbandry.

## 1 Introduction

The global imperative to curb the misuse of antibiotics represents a critical frontier in public health (1). The alarming rise in antibiotic resistance stems from the overuse of antibiotics in agriculture, which can render drugs ineffective (2). This makes research into alternatives to antibiotics particularly important. Meanwhile, the escalating climate crisis exacerbates another physiological challenge: oxidative stress (3). High temperatures and air pollution increase the production of harmful free radicals in animals and crops which always suffered from heat stress (4). This has led to a surge in demand for effective antioxidants to enhance the antioxidant capacity of biology. Natural antioxidants from plants have attracted significant attention not only for their role in mitigating oxidative damage but also for their potential antimicrobial properties (5, 6). Consequently, scientific focus is shifting towards sustainable and natural solutions. Hydroxytyrosol (HT), a potent phenolic compound primarily derived as a natural constituent and major metabolite of oleuropein found abundantly in olive fruits, leaves, and olive mill wastewater, which holds substantial promise as a functional natural feed additive in animal production due to its unique chemical structure and multifaceted biological activities (7). Characterized chemically by an ortho-dihydroxy (catechol) group and a phenolic hydroxyl group, HT exhibits exceptional free radical scavenging capacity, high antioxidant potency surpassing vitamin C and vitamin E in some assays, amphipathic properties enhancing bio-availability, and significant stability, enabling it to effectively quench reactive oxygen and nitrogen species, chelate pro-oxidant metal ions, and modulate critical cellular signaling pathways (8). Recent studies highlight HT multifaceted benefits in poultry production. Research demonstrated that dietary supplementation with HT in broiler diets significantly enhanced serum antioxidant capacity by increasing catalase (CAT) activity and reducing malondialdehyde (MDA) levels, while also modulating inflammatory responses via suppressing TLR4/NF-κB pathway expression in the jejunum (9, 10). Mechanistic investigations reveal that HT not only activates the Nrf2 antioxidant signaling pathway but also positively modulates gut microbiota, increasing beneficial *Lactobacillus* and *Firmicutes* while reducing *Bacteroidetes*, thereby boosting endogenous antioxidant enzyme production and mitigating oxidative damage. A significant focus of recent research is on mitigating intestinal oxidative damage in swine. Studies indicate that hydroxytyrosol (HT) can activate the PI3K/Akt-Nrf2 signaling pathway and promote mitophagy (the removal of damaged mitochondria) in porcine intestinal epithelial cells (IPEC-J2) (11). This dual activation enhances antioxidant defense system *in vivo*. It increases the activity of enzymes like catalase (CAT) and superoxide dismutase (SOD) while reducing markers of oxidative damage such as malondialdehyde (MDA) (12). Consequently, HT helps strengthen intestinal barrier integrity by boosting the expression of tight junction proteins such as ZO-1 and Occludin. Research models, such as those using diquat to induce oxidative stress, have shown that HT supplementation significantly alleviates intestinal damage and reduces serum markers of intestinal permeability like D-lactate and diamine oxidase (DAO) (13). While there are fewer studies reported in the field of livestock and poultry production. This is mainly due to a lack of understanding regarding the origin and synthesis methods of HT, as well as about its digestion, absorption, and metabolic pathways *in vivo*, along with its biological activities and modes of action in animals. Compared with most natural antioxidants, HT has a simpler production process and lower costs. Under the condition of the same antioxidant performance, HT is more suitable as an additive in animal husbandry. Therefore, this article aims to review the research progress of HT, summarize its sources, synthesis methods, digestion, absorption and metabolic pathways in animals, explain its main physiological functions, and its application prospects in livestock production, in order to develop a new natural green feed additive for animal husbandry.

## 2 Methods

### 2.1 Literature search strategy

A systematic literature search was performed to identify all relevant studies published in recent 10 years on HT, especially in the past 5 years. In the review, we added 71 references in total to strength. The following electronic databases were queried such as PubMed, Web of Science and Google Scholar. The search strategy utilized a combination keywords related to hydroxytyrosol, livestock, gut health, oxidative stress, liver protection and feed additive.

### 2.2 Study selection

Studies were included if they met the following criteria: firstly, the paper should be an original paper with a well-designed experiment and statistically significant results for data supporting. Secondly, the research had to focus on the application of hydroxytyrosol in livestock and poultry production. Thirdly, the research should be conducted within 5 years and the experiments should be reproducible. Conversely, studies were excluded if they related to human. Secondly, review articles were not searched for in this paper as references. Finally, articles with insufficient experimental design and sample size, or articles not in English were all not chose as references.

## 3 The sources, physicochemical characteristics and synthesis of hydroxytyrosol

**Figure 1:**
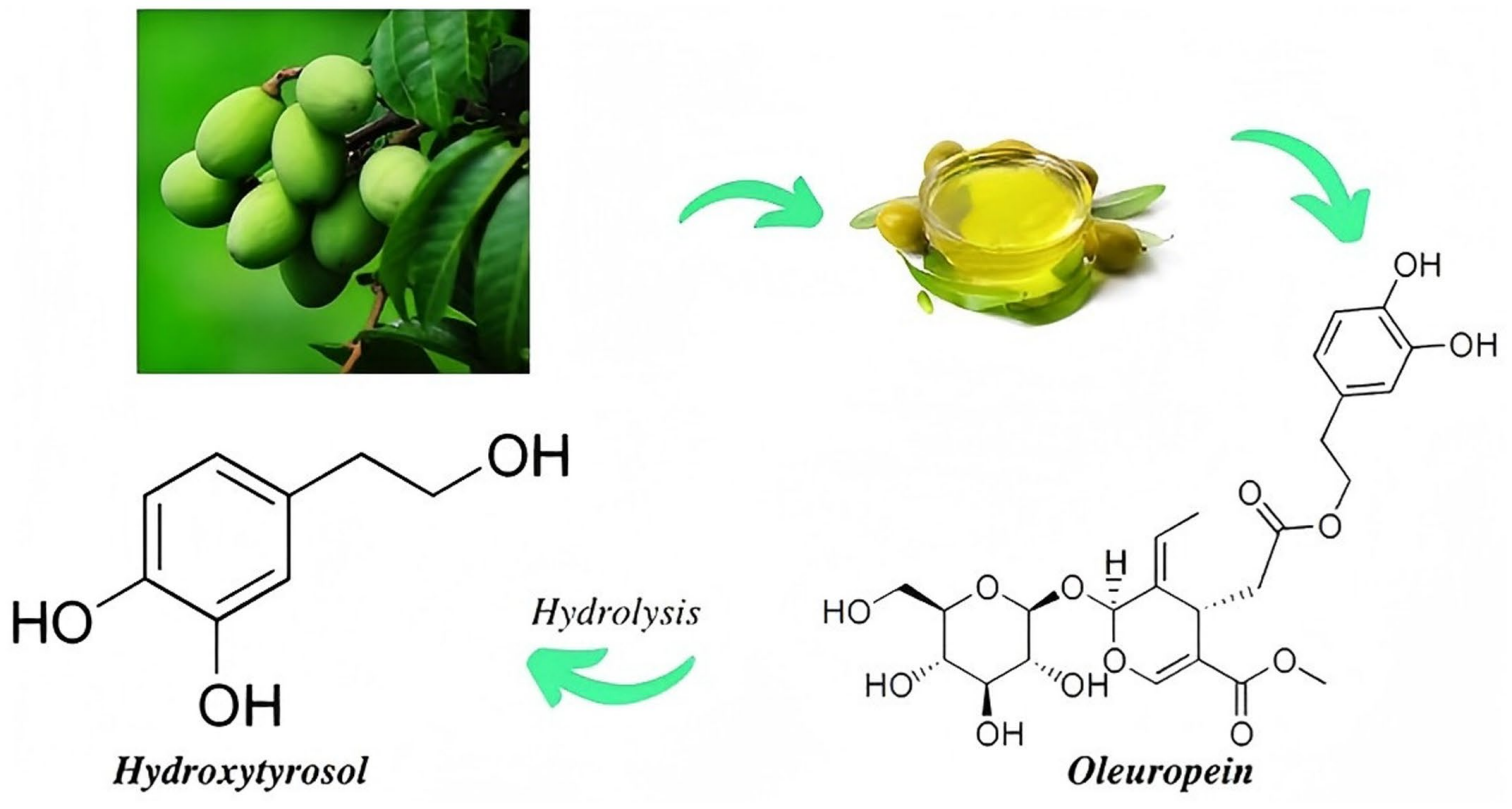
The source and structure of hydroxytyrosol.

For the sources of HT, it is a single-component phenolic substance mainly found in olive fruits, olive leaves and olive oil. It has been shown that HT in free form accounts for 6.0% of the total phenolic substances in olive oil (14). It is mainly produced by the hydrolysis of olives, which occurs during the ripening, storage and consumption of olives (15). Therefore, the HT concentration is affected by factors such as olive variety, maturity and processing technology (16). A small amount of HT is also present in red wine and white wine (17). The concentration ranges of HT from different sources are shown in Table 1. For the physicochemical characteristics of HT, it is an amphiphilic phenol (hydrophilic and lipophilic), its chemical name is 3,4-dihydroxyphenylethanol, its molecular formula is C_8_H_10_O_3_, and its relative molecular mass is 154.16. So its bio-availability is high (18). The source and structure of hydroxytyrosol was shown in Figure 1. For the synthesis of HT, currently, three main methods for the synthesis of HT have been studied, namely natural extraction, chemical synthesis and biosynthesis (19, 43). Among many chemical synthesis methods, the synthesis of HT using dopamine as raw material is the most successful method, but the process is complicated, the yield is insufficient, and the cost is high. The biosynthesis method does not require the use of any catalysts and harsh conditions. However, expensive substrates are the main bottleneck for this method to achieve industrial-scale production. Therefore, most industrial production currently uses natural extraction methods to extract HT from olive processing by-products and olive mill wastewater, which not only protects the ecological environment but also obtains high-value products. Considering the source, properties and synthesis method of HT, HT can be used as a new feed additive in animal nutrition.

**Table 1:** Concentration range of HT from different sources.

Source	HT concentration
White wine	1.5 ~ 2.7 mg/L
Red wine	2.0 ~ 3.9 mg/L
Aging red wine	25.0 mg/L
Virgin olive oil	0.01 ~ 0.021 mg/g
Olive leaf	10.0 ~ 17.0 mg/g

## 4 Digestion, absorption and metabolism of HT in animals

**Figure 2:**
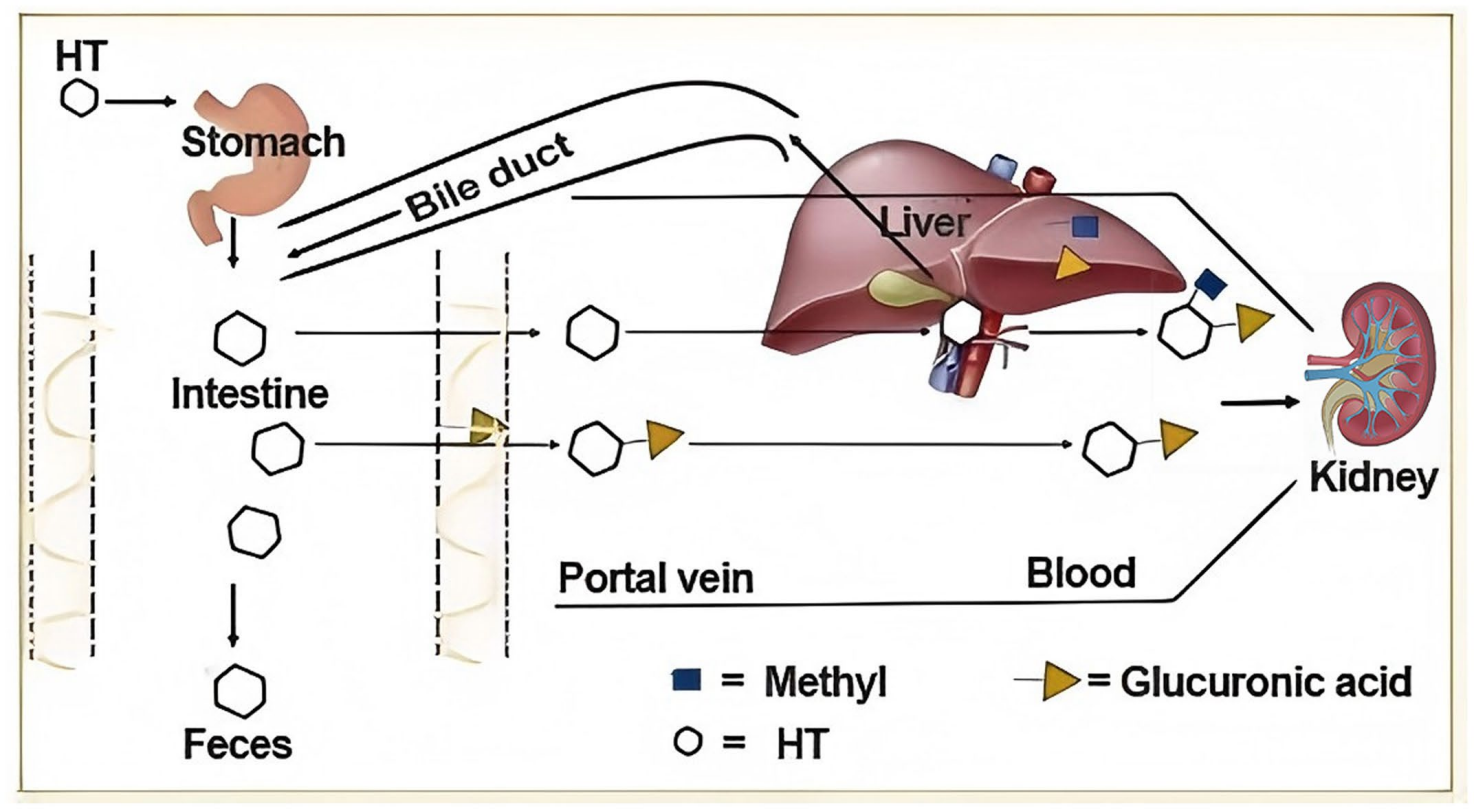
Summarize the metabolic pathways of HT *in vivo.* HT passes through the stomach, with a portion entering the systemic circulatory system through metabolism by the kidney and liver, while the other portion is absorbed and utilized through the intestine before being ultimately eliminated from the body.

Following ingestion as part of feed or a feed additive, hydroxytyrosol (HT) demonstrates considerable stability during digestion, resisting degradation in the acidic environment of the stomach (20). Upon reaching the small intestine, HT is efficiently absorbed primarily via passive diffusion across the enterocyte membrane, although involvement of specific transporters like monocarboxylate transporters (MCTs) may also facilitate its uptake, leading to rapid appearance in the bloodstream within minutes to hours (21). Once absorbed, HT undergoes extensive first-pass metabolism primarily in the intestinal mucosa and subsequently in the liver, where it is subjected to conjugation reactions, notably sulfation and glucuronidation, resulting in the formation of major metabolites such as hydroxytyrosol sulfate and hydroxytyrosol glucuronide conjugates (22). The absorption pathway is shown in Figure 2. These conjugated metabolites, while often less potent than the parent compound, still retain significant biological activity and represent the predominant forms circulating in the plasma and reaching systemic tissues. A portion of unabsorbed HT proceeds to the large intestine where it can be further metabolized by the gut microbiota, potentially undergoing transformations like dehydroxylation or other modifications, before eventual excretion of the remaining fraction and its metabolites occurs predominantly via the feces (for unabsorbed portions and microbially modified products). This efficient absorption and extensive conjugation pathway, generating bioactive metabolites, underpins the systemic delivery and physiological effects of HT and its derivatives throughout the animal body. Therefore, it is crucial to explore the bioavailability of exogenous HT in livestock models, which can provide a basis for the use of HT as a feed additive.

## 5 Biological functions of hydroxytyrosol

### 5.1 Anti-oxidant

Reactive Oxygen Species (ROS) are highly reactive molecules containing oxygen, generated through both normal cellular metabolism and exposure to external factors. This complex interplay of metabolic byproducts, enzymatic reactions, conversions, and environmental exposures constitutes the major pathways of ROS formation within biological systems. When the generation and clearance of ROS are not balanced, it often results in damaging to organs. Therefore, inhibiting ROS is fundamental to protecting cellular molecules (such as lipids, proteins or DNA) and avoiding the development of degenerative diseases (23, 24). The imbalance of ROS is due to the fact that the clearance capacity of the antioxidant system *in vivo* is less than the rate of ROS generation, which leads to a large accumulation of ROS and causes oxidative stress damage. At this time, antioxidants need to be supplemented *in vitro*. Its hydroxyl group (OH) has electron-donating ability at the adjacent position and can form stable hydrogen bonds with phenoxy groups, thereby reducing the generation of free radicals and inhibiting oxidative stress reactions (25–27). In swine, dietary HT supplementation significantly alleviates intestinal oxidative damage induced by herbicides like diquat. It directly activates the PI3K/Akt-Nrf2 signaling pathway in porcine intestinal epithelial cells (IPEC-J2), boosting the production of antioxidant enzymes to scavenge reactive oxygen species (ROS) (11). Concurrently, HT promotes mitophagy to remove damaged mitochondria, reducing ROS generation. The synergistic interaction between Nrf2 activation and mitophagy preserves gut barrier integrity, thereby improving nutrient absorption and growth efficiency (28). In broilers, HT (0.5 mg/g in feed) elevates serum antioxidant capacity, evidenced by increased catalase (CAT) activity and reduced malondialdehyde (MDA), a lipid peroxidation marker. It also down-regulates pro-inflammatory cytokines such as IL-1*β*, IL-6, TGF-*β* and suppresses the TLR4/NF-κB pathway in the jejunum, alleviating intestinal inflammation (29). These effects collectively enhance growth metrics: HT-supplemented broilers showed a 3.7% increase in final body weight and improved average daily feed intake (ADFI) compared to controls, nearing the performance of antibiotic-fed groups. HT can activate antioxidant enzymes that scavenge ROS and promote the expression of Nrf2, including *γ*-Glutamyl Cysteine Ligase (*γ*-GCL), heme oxygenase-1 (HO-1), NAD(P)H:quinone oxidoreductase (NQO1) and thioredoxin reductase (TrxR), inhibit the generation of ROS and vascular damage, and actively regulate the antioxidant defense system in vascular endothelial cells (30, 31). In addition to the above antioxidant mechanisms, HT can also directly scavenge free radicals and destroy free radical chain reactions, preventing the generation of a large number of new free radicals due to chain reactions, thereby reducing oxidative stress damage caused by excessive free radicals (32). HT can also bind to unstable metal ions in cells through hydroxyl groups, thereby protecting cells from DNA damage or cell apoptosis caused by oxidative stress (33).

### 5.2 Anti-inflammatory and antibacterial

In terms of anti-inflammation, Yao et al. (34) showed that the protective effect of HT on vascular endothelial cell inflammation is achieved through the PKM2 signaling pathway mediated by the SIRT6. In addition, studies have shown that HT has a therapeutic effect on COX-2-mediated inflammation and can be used in combination with traditional short-acting anti-inflammatory drugs (35). Scoditi et al. (36) found in an *in vitro* experiment that HT can inhibit the peroxisome proliferator-activated receptor *γ* (PPAR*γ*) signaling pathway in mouse 3 T3-L1 adipocytes, thereby showing anti-inflammatory activity. There are also relevant reports on *in vivo* studies. Liu et al. (37) added 50 mg/(kg·d) HT to the diet of C58BL/6 J mice for 8 weeks, it was shown that the levels of liver inflammatory markers including interleukin (IL)-1*β* and IL-6 were significantly decreased, the expression of tumor necrosis factor-*α* (TNF-*α*), IL-1*β*, Toll-like receptor 4 (TLR4) and phosphorylated phosphokinase (p-JNK) in the liver were reduced, meanwhile the release of lipopolysaccharide (LPS) in the blood was inhibited. Richard et al. (38) established an inflammatory model by stimulating mouse macrophages with LPS and evaluated the effects of HT on inflammatory mediators, cytokines and chemokines. The results showed that HT reduced the secretion of cytokines including IL-1*α*, IL-1*β*, IL-6, IL-12, TNF-*α* and decreased the gene expression of nitric oxide synthase (iNOS), IL-1*α*, CXCL10/IP-10, macrophage inflammatory protein-1*β* (MIP-1*β*), matrix metalloproteinase 9 (MMP-9), clarifying the molecular basis of HT in the treatment of inflammation. Zhang et al. (39) also determined the anti-inflammatory mechanism of HT by detecting the expression of iNOS, cyclooxygenase 2 (COX-2), the formation of TNF-*α* and the release of NO. The results showed that HT inhibited the expression of COX-2 gene in LPS-stimulated monocytes and significantly reduced the secretion of TNF-*α* and the release of NO, providing an effective theoretical basis for the treatment of inflammation. The previous research has found that the metabolite Tyr produced by the interaction of HT with intestinal microorganisms can prevent excessive production of nitric oxide (NO) by regulating p38 and ERK1/2MAPK, and concentrate it in the intestine, thereby significantly enhancing its protective activity against inflammation (40). In terms of antibacterial, a large number of studies have shown that HT has good antibacterial activity (41, 42). Rodríguez-Morató et al. (43) found that low concentrations of HT have antibacterial activity against respiratory and gastrointestinal pathogens such as *Vibrio enteritidis*, *Vibrio cholerae*, *Salmonella typhi*, *Haemophilus influenzae*, *Staphylococcus aureus*, and the inhibitory concentration is even lower than that of some antibiotics such as ampicillin. However, Medina-Martínez et al. (44) found that low concentrations of HT had a poor ability to inhibit bacterial growth, requiring 400 μg/mL to inhibit the growth of *Escherichia coli* strains. This phenomenon may be due to the oxidation of HT in nutrient-rich culture media, which weakened its antibacterial effect. Some studies have also shown that HT can effectively destroy fungal cell membranes and thus exhibit anti-fungal effects (45, 46). Therefore, HT has strong anti-inflammatory and antibacterial effects and can be used for diarrhea caused by intestinal inflammation in animals.

### 5.3 Lipid-lowering

Studies have reported that HT can reduce the differentiation and proliferation of adipocytes and reduce the number of lipid droplets in adipocytes (47–49). Garcia-Contreras et al. (50) added 1.5 mg/(kg·d) HT to the diet of pregnant sows. The results showed that HT did not affect the deposition of fetal fat, but significantly increased the synthesis of essential fatty acids in the fetus and significantly affected the ratio of ω-6 and ω-3 fatty acids, thereby improving the symptoms of intrauterine growth retardation in animals. This suggests that there are differences in the effects of HT on fat metabolism *in vivo*. In addition, experiments conducted by Echeverria et al. (51) on rats showed that the combined use of HT and eicosapentaenoic acid (EPA) can significantly reduce the occurrence of non-alcoholic fatty liver disease (NAFLD). The main effect of HT is that it enhances the antioxidant capacity and inhibits liver fatty degeneration. HT has antioxidant activity both *in vitro* and *in vivo*, not only can scavenge reactive oxygen free radicals, but also reduce the number of lipid droplets in hepatocytes, thereby reducing the accumulation of triglycerides in cells, and significantly reduce lipid synthesis (52). HT can also improve insulin resistance by regulating endoplasmic reticulum stress and prevent hepatic steatosis in diet-induced obese mice (53). In summary, on one hand HT can reduce the differentiation and proliferation of adipocytes to reduce the number of lipid droplets in adipocytes; on the other hand, it can reduce lipid synthesis and deposition by regulating the expression of inflammatory-related factors and strong antioxidant capacity. The mechanism action of HT under different conditions was shown in Table 2.

**Table 2:** The mechanism action of HT under different conditions.

Biological functions	Action target	Mechanism	References
Antioxidant	Antioxidant enzyme, ROS	Antioxidant enzymes 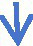 ROS production 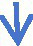	Servili et al. (27) Kouka et al. (26) Bertelli et al. (25)
Anti-inflammatory	IL-1*α*, IL-1*β*, IL-6, IL-12, TNF- *α*, CXCL10/IP-10, CCL2/MCP-1	IL-1α, IL-1β, IL-6, IL-12, TNF-α 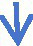 CXCL10/IP-10 CCL2/MCP-1 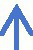	Richard et al. (38) Bedoya et al. (72)
Anti-bacteria	Bacterial and fungal membranes	Reduce the harmful bacteria in the gut such as *Escherichia coli* 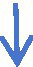	Medina-Martínez et al. (44) Aissa et al. (45) Diallinas et al. (46)
Lipid-lowering	Adipocytes and lipid droplets	Essential fatty acids 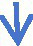 Liver fatty degeneration 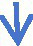	Lucas et al. (52) Drira and Sakamoto (47) Wang et al. (53)

## 6 Potential applications of hydroxytyrosol in livestock and poultry production

### 6.1 Application of HT in poultry production

Hydroxytyrosol (HT), a natural phenolic compound primarily derived from olives and olive oil, has garnered significant attention in poultry production due to its potent antioxidant, anti-inflammatory and antimicrobial properties. Recent research highlights its potential as a green feed additive alternative to antibiotics, particularly in broiler chickens (54). Studies demonstrated that dietary supplementation of HT in broiler diets significantly enhanced serum antioxidant capacity by increasing catalase (CAT) activity and reducing malondialdehyde (MDA) levels, thereby mitigating oxidative stress (55). Additionally, HT down-regulated key inflammatory pathways such as TLR4/NF-κB and reduces pro-inflammatory cytokines such as IL-1β, IL-6, TGF-β in intestinal mucosa, improving gut health (56). Although HT boosted average daily feed intake (ADFI), its effects on growth performance remain statistically insignificant compared to antibiotics, suggesting nuanced benefits (29). Emerging mechanisms reveal HT activated the Nrf2 antioxidant pathway and modulates gut microbiota, further enhancing antioxidant defenses and metabolic health (57). In summary, HT is a viable, natural strategy to promote poultry health in antibiotic-free farming, though further research is needed to optimize its application and assess long-term impacts.

### 6.2 Application of HT in swine industry

HT is also widely used as a feed additive in pigs. Recent research highlighted its efficacy in alleviating intestinal oxidative stress, a common issue in pigs caused by factors like weaning stress, mycotoxins, and high stocking densities (58). Studies demonstrated that dietary supplementation with HT significantly enhances antioxidant capacity by activating the PI3K/Akt-Nrf2 signaling pathway and promoting mitophagy in intestinal epithelial cells, thereby reducing oxidative damage and improving gut barrier function. For instance, HT was shown to increase serum levels of catalase (CAT) and superoxide dismutase (SOD) while decreasing malondialdehyde (MDA), a marker of lipid peroxidation (59). Additionally, HT modulated bile acid metabolism, further contributing to its antioxidative effects (13). In weaned piglets, HT supplementation improved growth performance, as evidenced by increased average daily gain (ADG) and reduced feed conversion ratio (F/G) (60). Moreover, HT enhanced intestinal health by up-regulating tight junction proteins such as ZO-1 and occludin, reducing pro-inflammatory cytokines such as IL-1β and IL-6 (61). Recent findings also indicated that HT benefits boar semen quality by improving gut microbiota and blood metabolome, increasing beneficial bacteria like *Bifidobacterium* and reducing harmful bacteria such as *Streptococcus* (62). Therefore, hydroxytyrosol had been widely used in swine industry. It is a new feed additive to replace antibiotics and has great potential in livestock production.

### 6.3 Application of HT in ruminants

Hydroxytyrosol (HT) as a natural polyphenol primarily derived from olives, shows emerging potential in ruminant nutrition, particularly for its antioxidant and anti-inflammatory benefits. However, direct evidence of its impact on methane emissions in cattle remains limited. Current research indicated that HT enhances antioxidant capacity in ruminants by activating the Nrf2 signaling pathway and improving gut microbiota composition such as increasing *Lactobacillus* and *Firmicutes* abundance, thereby reducing oxidative stress and supporting metabolic health (63). Although no studies explicitly link HT to methane reduction in ruminants, studies had shown that HT influences the rumen microbiota by selectively promoting beneficial bacteria and suppressing methanogenic archaea. Similarly, other plant-derived polyphenolic extracts have demonstrated positive effects on ruminal fermentation and degradability *in vitro*, supporting the potential of phenolic compounds to modulate microbial activity in ruminants (64). For example, studies indicated that HT supplementation increases the abundance of *Ruminococcaceae* and *Prevotella* which were the key taxa involved in fiber degradation and propionate production while reducing *Desulfovibrionaceae*, which is associated with sulfate reduction and methane formation. Shifts in microbial composition toward propionate-producing pathways to enhance energy harvest and reduce hydrogen availability for methanogenesis (65–67). Recent advancements in methane mitigation had focused on direct interventions like feed additives such as Plantain (68), but HT remains under-explored in this context. Future research should investigate the role of HT in modulating rumen fermentation and its synergy with established methane-reducing strategies. In conclusion, while hydroxytyrosol is not yet a proven methane-reducing agent in cattle, its strong antioxidant and microbiota-modulating effects position it as a promising supportive tool for improving ruminant health and potentially contributing to broader sustainable farming practices.

### 6.4 Application of HT in aquatic animals

**Figure 3:**
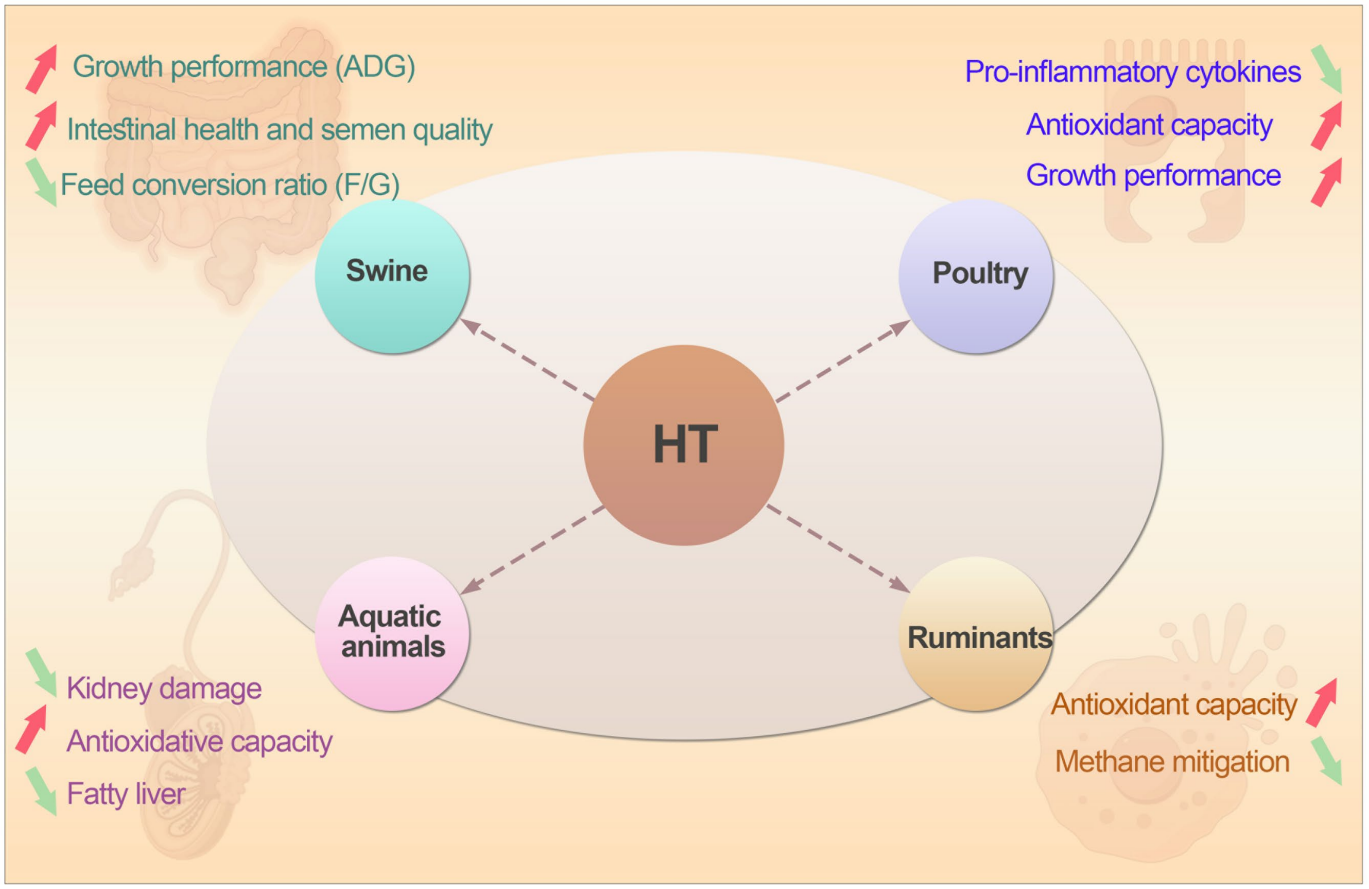
The summary of potential applications of hydroxytyrosol in swine, ruminants, poultry and aquatic animals.

Hydroxytyrosol (HT) has gained significant attention in aquaculture for its potent antioxidant, anti-inflammatory and metabolic regulatory properties. Recent research highlighted its multifaceted benefits across various aquatic species. In rainbow trout, HT supplementation effectively mitigated *Aeromonas hydrophila*-induced kidney damage by reducing pathological changes and modulating immune gene expression, thereby strengthening antioxidant defenses and immune response (69). In blunt snout bream, HT alleviated high-fat-diet-induced fatty liver by activating AMPK signaling, enhancing mitochondrial function and regulating lipid metabolism such as promoting fatty acid oxidation via PPAR-*α* pathways (70). Similarly, in gilthead sea bream, HT-rich extract reduced plasma free fatty acids and muscle lipid peroxidation, improved liver lipid metabolism and enhanced antioxidative capacity, demonstrating its role in optimizing lipid utilization and health under high-fat diets (71). Compared to its application in terrestrial species, HT is more easily utilized by aquatic organisms and increase the utilization rate. Therefore, hydroxytyrosol emerges as a promising natural additive in aquaculture, offering solutions to oxidative stress, infectious diseases and metabolic disorders like fatty liver. However, optimal dosing and species-specific responses require further investigation to maximize its benefits in sustainable aquaculture practices. The summary of potential applications of hydroxytyrosol in livestock and poultry production was shown in Figure 3.

## 7 Conclusions and perspectives

HT has unique advantages as an animal feed additive. First of all, HT has multiple physiological functions such as anti-oxidation, antibacterial, anti-inflammatory and lipid-lowering, it also has great potential in improving the intestinal health, protecting animal livers and promoting animal bone development in livestock and poultry. With its abundant sources and excellent properties, HT has great development value as a natural feed additive.

However, current research on HT is mainly focused on *in vitro* models and mouse models, there are few reports on its application in livestock and poultry production, the study of HT *in vivo* is even more important, as it will provide ample evidence for its application in animal husbandry. In actual production, the cost of HT purification is very high, so most HT products on the market are in the form of oily viscous liquids, and high-purity powder is rare. Therefore, when adding HT to the animal diet, the mixing procedure may not be uniform, making it difficult to achieve the expected feeding effect. The utilization of HT in livestock and poultry diets can be improved by making the feed into pellets. It is expected that technological advances may overcome the problems of high cost and uneven mixing.

In future studies, researchers should focus on elucidating the relationship between hydroxytyrosol and mitochondrial function. The optimal concentration, bio-availability and mechanism of action in different livestock and poultry diets also need further exploration. Through efficient purification technology, HT will have higher value in future commercial use, helping to reduce costs in livestock production and improve animal health.
